# Supplementary material for: Predicting need for heart failure advanced therapies using an interpretable tropical geometry-based fuzzy neural network
Source: PLoS One. 2023 Nov 28;18(11):e0295016. doi: 10.1371/journal.pone.0295016 (PMC10684094; doi:10.1371/journal.pone.0295016)
Supplement: S1 File — (DOCX) [file pone.0295016.s001.docx]

**Supplementary Materials: Predicting Need for Heart Failure Advanced Therapies using an Interpretable Tropical Geometry-based Fuzzy Neural Network**

Yufeng Zhang^1*^, Keith D. Aaronson^2^, Jonathan Gryak^3^, Emily Wittrup^1^, Cristian Minoccheri^1^ Jessica R. Golbus^2^, Kayvan Najarian^1,4,5^

**Affiliations**

1. Department of Computational Medicine and Bioinformatics, University of Michigan, Ann Arbor, United States
2. Division of Cardiovascular Medicine, Department of Internal Medicine, University of Michigan, Ann Arbor, United States
3. Department of Computer Science, Queens College, City University of New York, New York, United States
4. Michigan Institute for Data Science, University of Michigan, Ann Arbor, United States
5. Department of Electrical Engineering and Computer Science, University of Michigan, Ann Arbor, United States

**Table of Contents:**

- Page 2-3: Supplementary methods details
- Page 4-8: Supplemental Table S1-S3

**Training details for TGFNN.**

1. The data is split into training and testing dataset. The training dataset then is split into five folds for cross validation. The optimal model is obtained by conducting hyperparameter grid searches, which involve exploring various combinations of learning rate, regularization coefficients, the number of rules, and the stopping value for ϵ. This rigorous search enables the identification of the best set of hyperparameters that yield the most effective model. The algorithm is trained using the stochastic gradient descent method with the Adam optimizer. The weighted cross entropy loss function is employed to address data imbalance, alongside a regularization term to encourage concept and rule sparsity.
2. The number of total samples and positive samples for training, validation and test samples are listed in Table S3.
3. For every fold, a set of rules are extracted and then clustered by their correlations.
   - 1. Concepts with contributions less than 0.5 are removed.
     2. Rules with weight less than 0.5 are removed.
     3. Rules with correlation coefficients larger than 0.9 are removed.
4. Sets of rules from 5 models are then ensembled to initialize the model when it is retrained on the whole training dataset.
5. Hyperparamter search ranges

param_grid = {'min_epsilon': [x for x in **np**.linspace(0.4,0.9,6)],

'n_rules': [20,25,30],

'learning_rate': **loguniform**(5e-3, 1e-1),

'sparse_regu': [0],

'corr_regu': **loguniform**(1e-8, 1e-4),

}

**Hyperparameter Search range for other models**

- **XGBoost:**

param_grid = {'n_estimators': [**int**(x) for x in **np**.linspace(200, 2000, 10)],

*# Maximum tree depth for base learners*

'max_depth': [**int**(x) for x in **np**.linspace(2, 20, 10)],

*# Learning rate*

'eta': [x for x in **np**.linspace(0.1, 1, 10)],

}

- **SVM:**

param_grid = {'kernel': ['linear', 'rbf', 'poly', 'sigmoid'],

*# Regularization parameter.*

'C': **loguniform**(1e-1, 1e3),

*# Kernel coefficient for ‘rbf’, ‘poly’ and ‘sigmoid’.*

'gamma': **loguniform**(1e-4, 1e0)

}

- **Decision Tree**

param_grid = {

'max_depth': [**int**(x) for x in **np**.linspace(5, 25, 10)] + [None],

*# Minimum number of samples required to split a node*

'min_samples_split': [**int**(x) for x in **np**.linspace(2, 10, 9)]

*# Minimum decrease in impurity required for split to happen*

}

- **Random Forest**

param_grid = {'n_estimators': [**int**(x) for x in **np**.linspace(200, 1000, 5)],

*# Maximum number of levels in tree*

'max_depth': [**int**(x) for x in **np**.linspace(5, 25, 10)]+[None],

*# Number of features to consider at every split*

'min_samples_split': [**int**(x) for x in **np**.linspace(2, 10, 9)],

*# Minimum decrease in impurity required for split to happen*

'max_features': ['sqrt', 'log2'],

}

| Vital signs after admission | Features | Missing rate (%) | Units | Range for (+) class* | Range for (-) class^†^ |
| --- | --- | --- | --- | --- | --- |
|  | BMI | 0.00 | kg/m2 | 29.09 ± 6.16 | 30.83 ±7.65 |
|  | Heart Rate | 4.67 | bpm | 86.09 ± 18.85 | 86.15 ± 16.68 |
|  | Pulse Press | 0.00 | mmHg | 38.65 ± 11.87 | 48.47 ± 16.81 |
| Lab metabolites after admission | Albumin | 6.46 | g/dL | 3.95 ± 0.43 | 3.81 ± 0.41 |
|  | Uric Acid | 90.50 | mg/dL | 9.04 + 2.89 | 10.30 ± 3.10 |
|  | Hemoglobin | 0.00 | g/dl | 12.75 ± 1.94 | 12.17 ± 2.17 |
|  | Cholesterol | 86.25 | mg/dL | 137.77 ± 47.75 | 131.16 ± 42.79 |
|  | ALK | 8.98 | IU/L | 101.22 ± 52.53 | 113.06 ± 61.61 |
|  | ALT | 9.16 | IU/L | 94.22 ± 292.11 | 76.19 ± 293.64 |
|  | AST | 8.98 | IU/L | 76.5 ± 277.54 | 91.99 ± 403.18 |
|  | Bilirubin | 97.80 | mg/dL | 1.10 | 0.83 |
|  | CO2 | 0.36 | mmol/L | 27.16 ± 3.81 | 27.61 ± 3.88 |
|  | Hematocrit | 0.00 | % | 38.73 ± 5.36 | 37.15 ± 5.83 |
|  | Lymphocytes | 10.41 | % | 20.39 ± 8.87 | 20.25 ± 10.00 |
|  | Platelet | 0.36 | K/uL | 217.14 ± 83.53 | 213.94 ± 78.48 |
|  | Urea Nitrogen | 0.18 | mg/dL | 33.61 ± 19.03 | 32.26 ± 19.16 |
|  | White Blood Cell | 0.00 | K/uL | 8.50 ± 3.52 | 7.71 + 2.94 |
|  | Chloride | 0.18 | mmol/L | 100.62 ± 4.88 | 101.60 ± 5.79 |
| Echocardiogram | Mitral Regurgitation | 43.15 |  | 0 | 1 |

*Table S1: Clinical characteristic of patient encounters from Michigan Medicine (continuous features). The range statistics are presented as mean (standard deviation)*

**Patients who were urgent for advanced therapies; †* *Patients who were too well for advanced therapies*

*Abbreviations: BMI: body mass index; MAP: mean arterial pressure; ALK: Alkaline Phosphatase; ALT: Alanine Aminotransferase AST: Aspartate Aminotransferase LVIDd2D: left ventricular internal diameter end diastole in 2D; LVEF: left ventricular ejection fraction*

| Features | Overall prevalence (%) | Prevalence for (+) class* (%) | Prevalence for (-) class** (%) |
| --- | --- | --- | --- |
| Alcohol Abuse | 14.70 | 8.30 | 18.10 |
| Anemia (Blood Loss) | 14.50 | 9.30 | 17.30 |
| Cardiac Arrhythmias | 98.60 | 97.90 | 98.90 |
| Coagulopathy | 40.20 | 36.30 | 42.30 |
| Anemia (Iron Deficiency) | 43.10 | 34.20 | 47.80 |
| Depression | 41.50 | 28.50 | 48.40 |
| Diabetes (Complicated) | 42.90 | 33.20 | 48.10 |
| Diabetes (Uncomplicated) | 55.50 | 43.00 | 62.10 |
| Drug Abuse | 24.20 | 16.60 | 28.30 |
| Fluid Electrolyte Disorders | 78.30 | 67.40 | 84.10 |
| Hypertension (Complicated) | 68.00 | 59.10 | 72.80 |
| Hypertension (Uncomplicated) | 81.70 | 67.40 | 89.30 |
| Hypothyroidism | 26.60 | 28.50 | 25.50 |
| Lymphoma | 5.00 | 3.10 | 6.00 |
| Metastatic Cancer | 17.10 | 10.90 | 20.30 |
| Obesity | 53.50 | 46.10 | 57.40 |
| Other Neurological Disorders | 14.20 | 7.80 | 17.60 |
| Paralysis | 3.20 | 1.60 | 4.10 |
| Peptic Ulcer Disease - | 0.00 | 0.00 | 0.00 |
| Excluding Bleeding | 8.30 | 3.60 | 10.70 |
| Peripheral Vascular Disorders | 81.90 | 82.40 | 81.60 |
| Psychoses | 6.80 | 2.60 | 9.10 |
| Pulmonary Circulation Disorders | 70.60 | 65.80 | 73.10 |
| Rheumatoid Arthritis –  or Collagen Vascular Diseases | 7.90 | 9.30 | 7.10 |
| Solid Tumor –  Without Metastasis | 16.20 | 9.80 | 19.50 |
| Valvular Disease | 73.40 | 65.80 | 77.50 |
| Weight Loss | 33.60 | 22.80 | 39.30 |

*Table S2: Clinical characteristics of patient encounters from categorical variables. The range statistics are presented as mean (standard deviation)*

*All comorbidity features have no missing values.*

* *Patients who were urgent for advanced therapies; †* *Patients who were too well for advanced therapies*

|  | Total number of samples | Number of positive samples |
| --- | --- | --- |
| Training set | 368(7) | 126(3) |
| Validation set | 89(7) | 30(3) |
| Test set | 100 | 37 |

*Table S3: Average numbers of samples in the training, validation sets from 5 folds. The numbers are presented as mean (standard deviation).*
