# Supplementary material for: Predicting need for heart failure advanced therapies using an interpretable tropical geometry-based fuzzy neural network
Source: PLoS One. 2023 Nov 28;18(11):e0295016. doi: 10.1371/journal.pone.0295016 (PMC10684094; doi:10.1371/journal.pone.0295016)
Supplement: Attachment — Submitted filename: Response to Reviewers.docx [file pone.0295016.s002.docx]

Response to Reviewers

Dear reviewer,

Thank you for reviewing the manuscript **Predicting Need for Heart Failure Advanced Therapies using an Interpretable Tropical Geometry-based Fuzzy Neural Network**

for publication in PLOS One. We sincerely appreciate the time and effort taken to review our paper and provide insightful and constructive comments. We carefully considered and addressed the comments and suggestions provided by the reviewers. We appreciate all the comments, as it helped us to redesign the experiments and improve the current proposed algorithm. Our point-by-point response to the reviewers’ comments and concerns is provided below, along with a tracked changed version of the manuscript that highlights all changes. We also produce a final version of the revised manuscript, with all line numbers included below referring to the final untracked version.

**Response to Reviewer 1:**

(1)

Comment:

Authors should share the code and data of this work.

Response

Thank you for pointing out the data and code availability. The code now is available on <https://github.com/kayvanlabs/Generalized_fuzzy_neural_network_public>. However, for the data, this study involves human research participant data and due to privacy and data sensitivity, it cannot be publicly shared. For researchers who meet the criteria for access to confidential data, data are available from the University of Michigan’s Innovation Partnerships (UMIP) office upon request (contact innovationpartnerships@umich.edu)

(2)

Comment:

Figure 1 is so confused. Authors should update it.

Response

Thank you for your suggestions regarding the figure format. We have made updates to the figure to enhance its clarity in conveying the main framework and network structure. We changed the layout of the figure, add more details describing the experimental settings.

(3)

Comment:

It was suggested that MCC should be employed in this work.

Response

We appreciate your recommendation to include MCC as an additional evaluation metric. We have now computed MCC for all the models using both cross-validation and training-test schemes, and the results are included in the model performance tables.

(4)

Comment:

The language should be polished by native English speakers.

Response

Thanks for your suggestions. The manuscript has been revised by Emily Wittrup, a senior computational biologist and native English speaker. Additionally, Dr. Jessica Golbus and Dr. Keith Aaronson, both native speakers, have reviewed and approved the revised manuscript.

(5)

Comment:

Some efforts, such as 10.3389/fnins.2023.1197824, 10.1155/2022/9470683, can be discussed in this work.

Response

Thanks for your suggestions. These two papers have been a great source of inspiration in the field of bioinformatics. We have incorporated a discussion of these two papers into the introduction section of the manuscript.
